# Adaptation and validation of the Caregiver Burden Inventory in eating disorders

**DOI:** 10.1186/s40337-022-00560-7

**Published:** 2022-03-07

**Authors:** Sara Bertelli, Paolo Ferrara, Sharon Di Modica, Emilio Bergamelli, Orsola Gambini, Armando D’Agostino, Anne Destrebecq, Stefano Terzoni

**Affiliations:** 1 Department of Mental Health and Addiction, ASST Santi Paolo e Carlo, San Paolo Hospital, Milan, Italy; 2 Bachelor School of Nursing, ASST Santi Paolo e Carlo, San Paolo Hospital, Milan, Italy; 3 Department of Health Sciences, Università degli Studi di Milano, Milan, Italy; 4 CRC “Aldo Ravelli” for Neurotechnology and Experimental Brain Therapeutics, University of Milan Medical School, Milan, Italy; 5 Department of Biomedical Sciences for Health, Università degli Studi di Milano, Milan, Italy

**Keywords:** Caregiver Burden, Anorexia Nervosa, Bulimia Nervosa, Family support, Parental stress, First-degree relatives, Nursing, Assessment, Validation studies, Factor analysis

## Abstract

**Background:**

Living with people diagnosed with a mental disorder is known to increase the risk of developing high levels of so–called “caregiver burden” in informal caregivers. In–depth analysis of this phenomenon and specific assessment tools for caregivers of patients diagnosed with Eating Disorders (EDs) are lacking. In this study, we aimed to evaluate the psychometric properties of the Caregiver Burden Inventory in EDs and employ this adapted tool in this category of caregivers.

**Methods:**

A cross–sectional study was conducted in the Eating Disorders outpatient unit of an Italian University hospital. Face and content validity were investigated by calculating standard Content Validity Indices (CVI-I and CVI-S) after administering the Inventory to 6 expert nurses with at least 5 years of experience in mental health services assisting people diagnosed with Eating Disorders. Internal consistency was evaluated with Cronbach’s α coefficient for the overall scale and subscales. An exploratory factor analysis (EFA) was performed to explore latent constructs. The adapted CBI was then administered to 62 informal caregivers of ED patients.

**Results:**

The EFA yielded a 5–factor structure. The CVI-S was 97.2%; the Cronbach α coefficient was 0,90 (> 0.74 in each subscale). The median burden level in the experimental population was 40.0 [range = 21 to 54], in a theoretical range from 0 (no burden) to 96 (highest level of burden).

**Conclusion:**

The Caregiver Burden Inventory appears to be a valid and reliable instrument to assess caregiver burden in individuals diagnosed with Eating Disorders. Further research is needed to evaluate this tool’s efficiency in improving individually tailored interventions on families.

## Plain English summary

Eating disorders (EDs) are characterized by the development of abnormal eating habits, dysregulation of body weight and/or body image distortion. The pervasiveness of EDs may significantly affect the life of patients’ caregivers in terms of emotional load, termed Caregiver Burden (CB). This may impact the quality of life of the entire family and promote conflicts which may in turn exacerbate ED behaviours. Among several international assessment tools to measure CB, the Caregiver Burden Inventory (CBI) is perhaps the most widely employed. However, this tool has never been specifically tested in caregivers of ED patients. Here we show that CBI can adequately quantify burden in this category of caregivers. Systematic use of this instrument can support healthcare workers who aim to address the carer's discomfort and promote a focused monitoring of subjects with increased risk, in order to adequately plan targeted intervention programs.

## Introduction

Eating Disorders (EDs) are complex mental disorders defined by disturbed eating behaviours, distorted beliefs, and extreme concerns about food, eating and body image, shape or weight. The prevalence of these conditions is rising in many countries and recent studies suggest that EDs are not rare in the general population [1, 2]. Furthermore, EDs are associated with decreased quality of life, increased risk of depression and substance abuse [1]. The frequency and severity of clinical manifestations, and associated psychiatric and somatic comorbidities [3, 4] lead to high levels of disability and increased mortality rates [5]. Treatment approaches for EDs include behavioural interventions, psychological and pharmacological management and often require a multidisciplinary team composed of psychiatrists, physicians, psychologists, nurses, dietitians, and social workers. In this context, family involvement and informal caregiver support are known to have a fundamental role in building and maintaining patients’ therapeutic alliance and medical adherence [6, 7]. However, prolonged caregiving activity, albeit necessary to support patients as well as healthcare professionals, is known to affect the caregiver’s life substantially [8, 9]. The combination of physical, emotional, and social pressures can lead to a condition of intense distress and psychological discomfort called Caregiver Burden (CB), which can severely impact the quality of life of the entire family [10]. CB may fuel a disturbed relationship among family members, which may in turn exacerbate ED behaviours, as proposed by the recently revised Cognitive-Interpersonal Maintenance Model of Anorexia Nervosa (AN) [11, 12]. According to this model, emotional reactions with elevated anxiety and depression, psychological distress and dysfunctional responses to the illness, can cause divisions amongst family members, develop divergent forms of reactive expressed emotion and act as maintaining factors for the patient’s ED.

Although evidence on the burden of ED patients’ informal caregivers abounds, specific quantification instruments are lacking [13]. Several different assessment tools aiming to measure CB are available internationally, among which the Caregiver Burden Inventory (CBI) [14] is perhaps the most widely employed. Furthermore, the CBI is a multidimensional questionnaire used to quantify burden in different aspects of a caregiver's life, exploring five different dimensions: time dependence, developmental, physical, social, and emotional burden.

A large literature describes the use of the CBI to identify burden in caregivers of people affected by Alzheimer’s disease and related dementias [15] or with cancer, but this tool has never been employed in the assessment of ED patients’ caregivers. Therefore, the aims of this study were to assess the psychometric properties of an adapted version of the CBI and to employ it for the identification and quantification of burden in this category of caregivers.

## Methods

### Participants

Six expert nurses (with at least 5 years of previous assistance to people diagnosed with Eating Disorders) who work in the Department of Mental Health and Addiction of the San Paolo University Hospital in Milan, Italy were recruited to evaluate the scale’s face and content validity.

A non-probabilistic sample of informal caregivers was then recruited among ED patients who consecutively accessed the Eating Disorders clinic of the San Paolo University Hospital from May 1st to July 31st, 2019. This unit serves a population of approximately 350,000 inhabitants in the South of Milan. The caregiver could be any relative, partner or friend, excluding professional caretakers. Inclusion criteria were (i) a confirmed diagnosis of any ED formulated by an expert clinician (SB) according to Diagnostic and Statistical Manual of Mental Disorders, 5th edition [16] and (ii) at least one year of experience as caregiver for the recruited subject. A total of sixty–two caregivers accepted to participate (100% response rate). Forty–two (67,7%) were married, 17 (27,4%) separated and 3 (4,8%) unmarried. Forty (64,5%) carers had at least one other dependent child in addition to the one diagnosed with an ED. Thirty–eight (61,3%) caregivers were mothers, 23 (37,1%) were fathers and only one was a sister. The median duration of caregiving was 4,5 years [range = 3 to 5]. Of the patients diagnosed with an ED (58 females and 4 males), 40 (64,5%) were diagnosed with AN, 16 (25,8%) Bulimia Nervosa (BN), 4 (6,4%) with Unspecified Feeding and Eating Disorder and 2 (3,2%) with Binge-Eating Disorder.

### Questionnaire

In its original version, the Caregiver Burden Inventory is a 24-item, self-administered questionnaire [14]. Each of the 24 items allows a Likert-scale response ranging from 0 (“not at all relevant”) to 4 (“very relevant”), with a total score in the 0–96 range. Developed as a diagnostic tool for professional caregivers of confused and/or disoriented elderly people, the CBI yields five distinct sub-factors which address different aspects of caregiver burden: time dependence, developmental, physical, social, and emotional burden. Briefly, the time-dependent burden factor describes the burden due to restrictions on the caregiver's time; the developmental burden factor describes the caregiver’s feelings of being "off-time" in their development with respect to their peers; the physical burden factor describes caregivers' feelings of chronic fatigue and damage to physical health; the social burden factor describes caregivers' feelings of role conflict (a caregiver may argue with a spouse or with other family members over how to manage the care receiver's needs); the emotional burden factor describes caregivers' negative feelings toward their care receivers, which may result from the patient's unpredictable and often unfathomable behaviour.

The inventory showed good internal reliability, moderate intercorrelations of subscales, and high factor loadings, making it a practical tool for the assessment of caregiver burden [14].

### Procedures

Each participating nurse received a document with the definition of informal caregiver burden, information on the study purpose and the CBI. Nurses’ responses to the CBI were used to calculate the Content Validity Index of each single item (CVI-I) and of the whole scale (CVI-S). At the end of the questionnaire, two additional questions allowed the participant to communicate other relevant aspects that were not in the questionnaire and to report an overall opinion about clarity and exhaustiveness of the scale. Two items were modified according to these observations: Item 4 “I have to help my care receiver with many basic functions (dressing, washing, use of toilet)” was changed to “I have to help my care receiver with many basic functions (dressing, washing, use of toilet, meal assistance)”; item 5 “I don't have a minute's break from my caregiving chores” was changed to “I can’t get enough free time from my caring chores”.

The adapted tool was then administered to the sample of caregivers. Each recruited caregiver received a file including study information, consent form, the CBI and a questionnaire for the collection of socio-demographic data; all documents, independently filled out by each participant in a restricted area of the clinic, were then placed inside a sealed ballot box to guarantee full anonymity.

### Statistical analysis

An exploratory factor analysis (EFA) was carried out to identify the factor structure of the inventory. CBI-I and CBI-S were used to calculate the content validity of the scale; α Cronbach coefficient was calculated to evaluate the internal consistency. Factors were extracted with the principal component method, chosen according to Kaiser’s criteria (eigenvalue > 1) and rotated with the Varimax algorithm. Factor loading was assessed according to Stevens’ criterion.

Prior to the analysis, Kaiser–Meyer–Olkin (KMO) test and Bartlett’s test of Sphericity were employed to test the variables’ sampling adequacy. The KMO test measures sampling adequacy for each variable in the model and the complete model. This statistic is a measure of the proportion of variance that might be a common variance among variables, ie. which might be caused by underlying factors. Bartlett's test of Sphericity is used to confirm that the observed correlation matrix diverges significantly from the identity matrix, ie. a matrix in which all of the values along the diagonal are 1 and all other values are 0, making it suitable for a data reduction technique such as EFA.

Factor loadings were compared to Stevens’ cut–off, which is a value computed from the sample size, indicating the minimum acceptable factor loading. The median, first and third quartile scores and ranges of scores for each domain as well as the overall scale were verified. All analyses were conducted with SAS® 9 (SAS Inc., Cary, USA).

## Results

### Psychometric properties of the adapted CBI

The Kaiser-Mayer-Olkin test (0.74) and the Bartlett test correlation matrix (p < 0.01) confirmed adequacy of the data and sample size to carry out a factor analysis. Orthogonal factor rotation yielded clear separation of the five factors described in the original scale version (Table 1). All factor loadings were above Stevens’ cut-off and the model explained 63.9% of the total variance.

**Table 1:** CBI factor loadings

Item	Time-dependent burden	Developmental burden	Physical burden	Social burden	Emotional burden
1. My care receiver needs my help to perform many daily tasks	0.68				
2. My care receiver is dependent on me	0.77				
3. I have to maintain a high level of attention	0.74				
4. I have to help my care receiver with many basic functions (dressing it, washing it, use of toilets, meal assistance)	0.61				
5. I can’t get enough free time from my caring chores	0.74				
6. I feel I am missing out on life		0.71			
7. I wish I could escape from this situation		0.65			
8. My social life has suffered		0.58			
9. I feel emotionally drained due to caring for my care receiver		0.60			
10. I expected that things would be different at this point in my life		0.75			
11. I am not getting enough sleep			0.57		
12. My health has suffered			0.72		
13. Caregiving has made me physically sick			0.65		
14. I am physically tired			0.70		
15. I do not get along with other family members as well as I used to				0.66	
16. My caregiving efforts are not appreciated by others in my family				0.49	
17. I have had problems with my marriage				0.56	
18. I do not do as good a job at work as I used to				0.68	
19. I feel resentful of other relatives who could but do not help					0.51
20. I feel embarrassed about my care receiver’s condition					0.82
21. I feel ashamed of my care receiver					0.56
22. I resent my care receiver					0.61
23. I feel uncomfortable when I have friends over or when we go out					0.72
24. I feel angry about my interactions with my care receiver					0.50

Cronbach’s α was satisfactory (0.90); α coefficients were then calculated for each single subscale of CBI with values ranging from 0.74 and 0.91. According to the correlation table, no item reversing was necessary compared to the original scale version. The content validity index of the instrument (CVI-S) was 97.2% (CVI-I always ≥ 0.80).

### Burden in ED caregivers

The sample’s median burden score was 40.0 [range = 21 to 54], in a theoretical range from 0 (no burden level) to 96 (maximum burden level). Table 2 shows the burden observed for each subscale.

**Table 2:** Median CB scores in the subscales

Subscales	Me [range]	Theoretical range
Time-dependent burden	9.0 [3 to 11]	0 to 20
Developmental burden	12.0 [6 to 17]	0 to 20
Physical burden	6.5 [4 to 10]	0 to 16
Social burden	7.0 [5 to 11]	0 to 20
Emotional burden	6.0 [2 to 8]	0 to 20

## Discussion

The modified CBI demonstrated good psychometric properties to evaluate Caregiver Burden in patients diagnosed with an ED. The CVI-S score obtained by interviewing the expert nurses panel confirmed content validity of the scale, which was also found to be simple, clear and understandable. As its original version, the modified CBI provides a multidimensional view of burden and identifies the same subdomains through its subscales in EDs. Specifically, the Time-dependent Burden factor may reflect the caregiver’s time consumption in assisting patients across meal times and vigilant monitoring of eating and maladaptive behaviour (elimination, physical overactivity) on the caregiver's time. The Developmental Burden factor may reflect caregivers' feelings of slower and somewhat faulty development compared to their peers. Onset of EDs often occurs during early adolescence and may have a substantial impact over the life course of a caregiver. Although specific research on caregivers of ED patients is lacking, the Physical Burden factor reflects subjective complaints regarding physical health, on which informal caregiving towards dependent friends or family members is known to have a detrimental influence. The Social Burden factor may reflect the conflicts emerging with other relatives and friends over the best way to address the receiver’s needs. Indeed, individuals with EDs often elicit contradictory feelings on the extent to which support and limitations to maladaptive behaviour should be enforced by the informal caregiver. The Emotional Burden factor describes caregivers' negative feelings, which may result from the patient's unpredictable and often unfathomable behaviour.

The acceptable values of Cronbach’s α—for the scale and for each domain—confirmed the internal consistency of the adapted CBI. Indeed, only one previous study on the Italian version of the original CBI had identified four factors due to the conflation of the Developmental and Physical burden dimensions [15].

In our clinical sample, caregiver burden appeared to primarily reflect a reduction of personal time and development. Indeed, vigilant monitoring of eating and maladaptive behaviour (elimination, physical overactivity) and direct assistance across meal times may significantly impact caregivers’ everyday life. Further research is needed to establish whether these aspects vary with the severity and stage of illness, and if they can be modulated with tailored interventions.

To the best of our knowledge, no previous studies used the CBI to quantify burden in caregivers of individuals with an ED. The eating disorders symptom impact scale (EDSIS), a 24-item self-report questionnaire is currently only available in English [17] and Spanish [18]. The Family Problems Questionnaire [19] is a self-report measure with a a good level of reliability that has been employed to compare burden in families of AN and BN [20]. However, lack of use outside of Italy limits the possibility of comparing findings with those reported from international groups.

Although developed for caregivers of elderly patients, the CBI has also extensively been used to assess caregiver burden in parents of children with metabolic [21] and neurodevelopmental [22, 23] disturbances, and in families of young people presenting with First Episode Psychosis [24]. Our study confirms the possibility of employing this tool in caregivers of relatively young patients with psychiatric disturbances, perhaps suggesting the possibility of future comparative studies across clinical samples. Indeed, CB is known to be similar in the families of individuals with ED and psychosis [25], although some findings suggest the impact of caregiving for patients with EDs may be even higher than those with Schizophrenia and Depression [26]. Previous research on CB with ED patients employed the Depression Anxiety Stress Scales (DASS) or questionnaires based on the related construct of Expressed Emotion within the patient’s family [13]. However, these instruments do not specifically address the construct of CB and are likely to measure related, albeit different characteristics of caregivers’ distress. Disentanglement of these constructs is likely to clarify the mechanisms that lead caregiver distress to maintain the illness [12]. Indeed, novel approaches to the treatment of EDs centralize psychoeducational interventions on caregivers to moderate emotionally driven responses such as criticism, overprotection, accommodating and/or enabling behaviours that have been shown to maintain the illness [27]. Our findings suggest that nurses and clinicians should pay special attention to caregiver burden and the administration of the CBI alone could be a practical choice to identify contributing factors. However, future research is needed to identify a CBI cut-off score for further assessment and intervention.

### Limitations

This study aimed to offer a preliminary overview of the CB phenomenon in a specialized outpatient unit. However, the reduced sample size should certainly be considered the main limitation and a larger, possibly multicentric study is necessary to confirm our findings. Nonetheless, the significant levels of CB detected in a median 4.5 years of caregiving suggest the relevance of the phenomenon and the need to address it. Another limitation was the lack of a standardized interview to exclude psychiatric diagnoses in caregivers. Future studies are needed to clarify the relationship between CB and the socio-demographic and psychological characteristics of patients’ caregivers. A more thorough understanding of this connection is fundamental to the implementation of a preventive intervention path.

## Conclusions

The study results support the usefulness of CBI for the assessment of caregiver burden in patients diagnosed with EDs. The systematic use of this instrument can support physicians and nurses in assessing the carer's discomfort and a focused monitoring of subjects with increased risk, to adequately plan targeted intervention programs.

Future research should address whether early identification of burden and distress can be used to shape novel interventions with an impact on the psychopathological mechanisms underlying EDs.

## Data Availability

The datasets used and/or analysed during the current study available from the corresponding author on reasonable request.

## Abbreviations

ED: Eating disorders
EFA: Exploratory factor analysis
CB: Caregiver Burden
CBI: Caregiver Burden Inventory
KMO: Kaiser–Meyer–Olkin
